# Why Are New Tobacco Control Interventions Needed?

**DOI:** 10.3390/ijerph15040658

**Published:** 2018-04-02

**Authors:** Gera E. Nagelhout, Lucy Popova, Mirte A. G. Kuipers

**Affiliations:** 1 IVO Addiction Research Institute, 2595 AA The Hague, The Netherlands; 2 Department of Health Promotion and Department of Family Medicine, Maastricht University (CAPHRI), 6200 MD Maastricht, The Netherlands; 3 Division of Health Promotion and Behavior, School of Public Health, Georgia State University, Atlanta, GA 30302, USA; lpopova1@gsu.edu; 4 Department of Public Health, Academic Medical Center, University of Amsterdam, 1100 DD Amsterdam, The Netherlands; m.a.kuipers@amc.uva.nl

It has been known for years which policies and interventions work to decrease tobacco use in the population [1,2]. The almost worldwide ratification of the Framework Convention on Tobacco Control (FCTC) [3] reflects the consensus on strong government action being crucial in effectively preventing and reducing tobacco use, for example, by regularly increasing tobacco taxes while providing free cessation support to those who have trouble quitting smoking. Still, many researchers focus on developing and evaluating new tobacco control interventions. Why are these new interventions needed? As the research papers and protocols in this special issue of the International Journal of Environmental Research and Public Health illustrate, the answer to this question is three-fold.

First, while overall rates of smoking are decreasing, disparities in tobacco use and secondhand smoke exposure persist, with certain vulnerable populations benefiting less from the existing interventions. Several of the publications in this special issue focus on reducing tobacco use among vulnerable populations: people with severe mental illness [4], people living with Human Immunodeficiency Virus (HIV) [5], and Indigenous populations [6,7]. Smoking prevalence rates are two to four times higher [4,5,6,7] in these groups than in the general population. Therefore, it is crucial that future tobacco control efforts effectively reach these groups. In a systematic review, Stevenson and colleagues concluded that cultural aspects are important for smokefree home interventions for Indigenous groups [7]. Bar-Zeev et al. improved the cultural responsiveness of a tailored intervention for smoking cessation care in pregnant Aboriginal women [6]. For different vulnerable groups, similar efforts to tailor interventions are described in study protocols by Lawn et al. [4] and Bell et al. [5]. While FCTC Article 4 [3] called for political commitment to socially and culturally appropriate tobacco control programs for Indigenous populations, this seems important for a range of vulnerable groups. Two other publications in this special issue examined the differences in perceptions of product and packaging policies between low socioeconomic status (SES) and high SES individuals [8,9]. Although smoking rates among low SES groups are generally not as high as in the vulnerable populations discussed above [10], an increasingly large share of the total smoking population is of low SES [11]. In this special issue, low SES groups showed more positive attitudes towards further tobacco product legislation (e.g., restricting flavored cigarettes) [8], while they were less likely to find quitting tips messages on cigarette package inserts helpful [9] than their high-SES counterparts.

Second, there is a need to keep up with new technologies that some people nowadays prefer to use over older technologies. New technologies for tobacco control interventions that are the topic of publications in this special issue are: smoking cessation apps or programs [4,12], personal carbon monoxide monitors [13], and electronic cigarettes [5,14]. All of these publications are study protocols, needs assessments, or pilot trials, so no definitive conclusions can be drawn from them. We do know from previous studies that existing smoking cessation apps are of variable quality [15,16,17], so we welcome the efforts to describe intervention features and development in study protocols and to rigorously pilot test interventions before implementation, as is good scientific practice. Two studies in this special issue focus on electronic cigarettes. Bell et al. [5] propose a feasibility study of using electronic cigarettes as a harm-reduction tool for people living with HIV who smoke. Russo et al. [14] demonstrate that electronic cigarettes might help prevent weight gain following smoking cessation. While there is mixed evidence from clinical trials and population-level studies on whether electronic cigarettes help smoking cessation, there might be a role for electronic cigarettes in certain cessation contexts.

Third, existing policy measures or interventions that enjoy popular and political support can be extended to new products, locations, and populations. The articles in this special issue examined a variety of such interventions. Schmidt and colleagues [8] evaluated the level of support among the U.S. public for some of such novel interventions, and found that the majority supported regulations to reduce nicotine in cigarettes, and ban candy or fruit-flavored little cigars and electronic cigarettes. Scheffers-van Schayck et al. [18] describe a protocol for a randomized controlled trial and an implementation study aimed at proactive smoking cessation counseling for smoking parents. Several studies gave indications for the effectiveness of the various interventions, such as extending smokefree policies in schools to include outdoor spaces in the Netherlands [19], cigarette package inserts in Canada [9], and health warnings in electronic cigarette ads in the U.S. [20]. For example, the study by Thrasher et al. [9] found that about eight in ten smokers identified at least one general cessation benefit message on a cigarette package insert to be motivating or a quitting tip message to be helpful.

In conclusion, while tried-and-true tobacco control interventions should continue to be implemented, novel tobacco control interventions provide new opportunities to reach vulnerable populations for whom the rates of tobacco use have been slower to decline, to utilize new technologies, and to adapt existing interventions in promising new ways. Finally, what also needs to be considered is that tobacco control is a unique area in which efforts at combatting disease and promoting health are met with active resistance from the industry. The tobacco industry has been called “the vector of disease” [21] and is constantly developing new products and new ways to resist and circumvent existing regulations, policies, and interventions. Because of that, vigilance and new developments are needed from the public health sector. Therefore, we recommend that researchers and practitioners keep developing and evaluating new tobacco control interventions.
